# Resolving plasmid-encoded carbapenem resistance dynamics and reservoirs in a hospital setting through nanopore sequencing

**DOI:** 10.1099/mgen.0.001644

**Published:** 2026-02-12

**Authors:** Ela Sauerborn, Rhys T. White, Anna-Lena Kalteis, Daniel Gygax, Ebenezer Foster-Nyarko, Nina Wantia, Friedemann Gebhardt, Lara Urban

**Affiliations:** 1 Helmholtz AI-Institute, Helmholtz Center Munich, Munich, Germany; 2 Helmholtz Pioneer Campus, Helmholtz Center Munich, Munich, Germany; 3 Department of Preclinical Medicine, Institute of Medical Microbiology, Immunology and Hygiene, Technical University of Munich, Munich, Germany; 4 New Zealand Institute for Public Health and Forensic Science, Health Security, Porirua, New Zealand; 5 Technical University of Munich, TUM School of Medicine and Health, TUM University Hospital, Munich, Germany; 6 RIFCON GmbH, Hirschberg an der Bergstraße, Germany; 7 Department of Infection Biology, London School of Hygiene & Tropical Medicine, London, UK; 8 Institute for Food Safety and Hygiene, University of Zurich, Zurich, Switzerland

**Keywords:** carbapenem resistance reservoirs, clinical resistance surveillance, long-read nanopore sequencing, strain and plasmid clustering

## Abstract

The growing resistance of *Enterobacterales* to last-resort antibiotics such as carbapenems puts a significant burden on healthcare systems, also due to plasmids driving a rapid spread of carbapenem resistance. We here evaluate the use of long-read nanopore sequencing to investigate carbapenem resistance dynamics and the role of plasmid transfers and environmental reservoirs in the hospital setting. Over 13 months, routine clinical diagnostics identified recurring isolates of carbapenem-resistant *Citrobacter* species carrying *Klebsiella pneumoniae* carbapenemases (KPCs) and/or OXA-48-like carbapenemases from patient screening and hospital drain samples. While routine diagnostic approaches provided limited insights into the carbapenem resistance dynamics, we show that near-complete *de novo* assembly of chromosomes and plasmids by long-read nanopore sequencing allowed for high-resolution strain identification, plasmid profiling, and antibiotic resistance gene detection. Notably, genomically nearly indistinguishable *Citrobacter freundii* of the high-risk sequence type ST91 genomes were recovered from screening samples collected in the same hospital room 1 year apart. We further provide evidence of a KPC-2-encoding IncN plasmid that is likely to have spread across bacterial species and between patient and drain isolates, which emphasizes the role of contaminated drains in the persistence and dissemination of antimicrobial resistance within the hospital environment. Overall, this study demonstrates the value of long-read nanopore sequencing for uncovering the complex dynamics of carbapenem resistance spread and persistence in the hospital setting and its potential implications for infection prevention and control.

**Impact Statement**

This study demonstrates how long-read nanopore sequencing can resolve the complex dynamics of plasmid-mediated antimicrobial resistance in clinical and environmental samples within the hospital setting. By linking patient- and drain-derived isolates through near-complete *de novo* assemblies, we reveal hidden reservoirs and dynamics behind the persistence of carbapenem resistance over extended time periods. This work shows how long-read sequencing approaches can uncover resistance dynamics that are missed using standard diagnostic methods, with implications for infection control and surveillance.

## Data Summary

The study sequences are available at the National Center for Biotechnology Information (NCBI) under BioProject accession number PRJNA1297122. The raw sequence read data is available at the NCBI Sequence Read Archive (SRA) (https://www.ncbi.nlm.nih.gov/sra) under accession numbers SRR34727947–59. The chromosomal assemblies of all ST91 strains are available at the NCBI GenBank under the Biosample accession numbers SAMN50449475–80. All IncN plasmid sequences were made publicly available in FigShare (https://doi.org/10.6084/m9.figshare.30519272). All other supporting data are provided in the article and supplementary data files.

## Introduction

Antimicrobial resistance, particularly to last-resort antibiotics such as carbapenems, is a growing health concern [1,3]. Infections with carbapenem-resistant *Enterobacterales* (CRE), including *Citrobacter* spp., present significant clinical challenges due to limited therapeutic options, leading to delayed or ineffective treatment and increased morbidity and mortality [1]. The dissemination of carbapenem resistance is primarily driven by horizontal gene transfer via plasmids [4,8]. This mechanism enables the rapid spread of resistance across bacterial species boundaries, complicating efforts to control the plasmid-mediated resistance spread in healthcare settings [6,7, 9]. Understanding plasmid-encoded resistance dynamics is therefore crucial for developing effective containment strategies.

Environmental reservoirs of CREs within healthcare facilities serve as persistent sources of CRE transmission and amplification. Hospital sink and shower drains have been identified as particularly relevant reservoirs, providing optimal conditions for CRE survival and proliferation [10,12]. In clinical settings, the impact of carbapenem resistance spread is largely determined by how quickly it is detected, as this directly affects the speed and effectiveness of the infection prevention and control (IPC) response [13]. Rapid and accurate genomic surveillance and plasmid characterization of both patient and environmental samples provide more insight into the spread and persistence mechanisms of carbapenem resistance [7,9, 14,16]. However, many clinical microbiology laboratories lack in-house genomic capabilities and instead rely on phenotypic testing methods that cannot resolve resistance mechanisms at the genetic level, or on external sequencing services that provide results too slowly to inform rapid IPC responses. On-site nanopore sequencing offers advantages for real-time genomic surveillance in the clinical setting. Its capacity for long-read sequencing enables the resolution of complex genomic structures, including complete plasmid sequences [17], while its rapid turnaround time supports timely IPC decision-making. These capabilities make nanopore sequencing particularly valuable for disentangling complex epidemiological relationships in healthcare-associated outbreaks [17,21].

In this study, we conducted a retrospective genomic investigation of a carbapenem-resistant *Citrobacter* spp. cluster in an internal medicine ward using whole-genome sequencing. Over a 13-month period, routine laboratory diagnostics detected *Klebsiella pneumoniae* carbapenemase (KPC)-producing and/or OXA-48-like carbapenemase-producing *Citrobacter* spp. in patient screening and environmental samples from sink and shower drains. We here show that conventional diagnostic approaches based on phenotypic testing and lateral flow assays could not sufficiently resolve the dynamics behind this cluster of carbapenem-resistant *Citrobacter* spp. In contrast, thorough genomic data analysis allowed us to characterize complex carbapenem resistance dynamics and identify critical environmental reservoirs contributing to persistence and transmission within the hospital setting.

## Methods

### Sample collection

From February 2024 to March 2025, recurring carbapenem-resistant *Citrobacter* spp. carrying KPC and/or OXA-48-like carbapenemases were repeatedly detected in patient screening and environmental samples from an internal medicine ward at the Technical University of Munich (TUM) Hospital in Munich, Germany. Relevant isolates were detected in ten screening samples from patients (rectal swabs and one urine sample) and in three environmental (sink and shower drains) samples. Rectal swab collection for CRE detection is routinely conducted as part of the admission screening at the hospital. Briefly, rectal swabs (Copan Diagnostics, Brescia, Italy) were collected by insertion to a depth of 2–3 cm with threefold rotation. One mid-stream urine sample was included in this study due to the phenotypic detection of carbapenem-resistant *Citrobacter* spp. (see below for details on phenotypic tests). The ten patient samples were obtained from different individuals. These samples represent a subset of 275 patient samples in total that were obtained during the 13-month study period as part of routine carbapenem-resistance surveillance. The samples were plated on BD® MacConkey (Becton Dickinson GmbH, Heidelberg, Germany), and Thermo Scientific™ Brilliance™ extended-spectrum beta-lactamase (ESBL) agar plates and incubated at 37 °C for 20–24 h.

Environmental drain sample collection was conducted in October 2024 on the ward, from rooms with relevant patient occupancy in this study (out of eleven patient rooms on the ward). The environmental samples included in this study correspond to 13 environmental samples in total, taken from all rooms with patient occupancy in the ward in October 2024. Room A was a one-bed and room D a two-bed patient room, and rooms B and C were three-bed patient rooms. At the time of sampling, the sanitary facilities were cleaned as part of the daily routine in accordance with the hospital’s internal cleaning and disinfection protocol by using a limescale-removing sanitary cleaner (Milizid®, DR. SCHNELL GmbH and Co. KGaA, Munich, Germany). No specific treatment of the drains was carried out prior to drain sampling.

For sample collection, a 50 ml bladder syringe was fitted with a suction catheter and inserted into each drain. The system was repeatedly aspirated and flushed to mix the contents, and 20 ml of this mixture was drawn from the drain and transferred into tryptic soy broth enriched with disinhibitor to deactivate residual disinfectants (Thermo Scientific™) for incubation. After 20–24 h, 100 µl of the broth was plated out on BD® Columbia Blood, MacConkey (Becton Dickinson GmbH), and Thermo Scientific™ Brilliance™ ESBL agar plates; if no growth was detected in the broth after 48 h, the plating was repeated. Environmental samples showing no growth in the broths or on the plates thereafter were declared negative.

### Established diagnostics

For established laboratory diagnostics, species identification was conducted from a single colony-forming unit (CFU) of bacterial isolates with growth on ESBL agar plates using MALDI-TOF MS (Bruker Daltonics GmbH, Bremen, Germany, MBT Compass 4.1, MBT Compass Reference Library 2023), as per the manufacturer’s instructions. Phenotypic antibiotic susceptibility testing was performed using VITEK 2 (BioMérieux, Marcy l’Etoile, France) on pure subcultures. For this, up to three bacterial CFU were transferred to a saline tube to generate a homogenous suspension with a density equivalent to 0.5 McFarland. Subsequently, minimum inhibitory concentrations (in mg l^−1^) of the bacterial isolates were determined using the VITEK 2 gram-negative (AST-GN69) card, and the carbapenem susceptibility results were interpreted according to the European Committee on Antimicrobial Susceptibility Testing (EUCAST) guidelines [22]. Carbapenemases were identified using a multiplex immunochromatography assay consisting of lateral flow assays (O.K.N.V.I Resist-5) that detects the presence of KPC, OXA-48-like, NDM, VIM and IMP carbapenemases, according to the manufacturer’s instructions. After completing these routine diagnostic steps, five to ten CFU of each isolate were stored at −80 °C in glycerol stocks for future use.

### DNA extraction and nanopore sequencing

Stored isolates were grown overnight at 37 °C on BD® Columbia Blood Agar plates. Per isolate, DNA was extracted in a spin-column-based DNA purification approach. For this, 10–20 CFU of overnight bacterial cultures were resuspended in 1 ml PBS. A 100 µl aliquot of this suspension was transferred to a fresh 1.5 ml Eppendorf tube and adjusted to 1 ml with PBS, mixed gently and centrifuged at 12,000 ***g*** for 2 min. The supernatant was discarded, and genomic DNA was extracted from the resulting pellet with the Qiagen DNeasy Blood and Tissue kit (Qiagen, Hilden, Germany), following the manufacturer’s Gram-negative bacteria protocol [23]. We added 40 µl of proteinase K and extended the lysis incubation to 2 h to increase the yield and purity of the DNA extracts.

The resulting DNA extracts were quantified using a Qubit 4 Fluorometer based on DNA-specific fluorescent dyes (dsDNA HS kit) and by NanoDrop One Spectrophotometer measurements of total nucleic acids based on UV-absorbance at 260 nm. In a subset of DNA extracts, we observed a discrepancy between Qubit and NanoDrop DNA concentration measurements (Table S1, available in the online Supplementary Material), indicating the presence of RNA (Table S1), which can impact library preparation and contribute to the background sequencing signal. Indeed, high levels of failed sequencing reads (Fig. S1a) and unavailable nanopores (Fig. S1c) when sequencing these extracts were detected, preventing any further robust downstream analysis due to low sequencing output. We therefore treated these DNA extracts with high RNA content post hoc by adding 4 µl of DNase-free RNase (10 mg ml^−1^, Thermo Fisher Scientific, Waltham, USA) directly to 50 µl of the DNA extracts, followed by vortex mixing and a 15-min incubation step at room temperature. As DNA yield was reduced after RNase treatment (Table S1), the resulting DNA extracts were further purified and concentrated with the Zymo DNA Clean and Concentrator-5 kit (Zymo Research, Irvine, USA) according to the manufacturer’s instructions. The post hoc RNase treatment substantially removed residual RNA and resulted in improved sequencing outcomes (Table S1, Fig. S1b, d). However, as it also reduced the DNA yield and purity (Table S1), we only recommend its post hoc application to non-precious DNA extracts. Based on these observations, an RNase step was incorporated into the protocol of subsequent DNA extractions after the proteinase K incubation step, following the manufacturer’s instructions (Qiagen).

Nanopore sequencing libraries of all DNA extracts were generated using the SQK-RBK114 Rapid Barcoding Kit and sequenced on R10.4.1 flow cells for 16–20 h using an Oxford Nanopore Technologies MinION MK1d device. We used two barcodes per DNA extract to account for imbalanced sequencing outputs. Eighty to two hundred nanograms of DNA in 10 µl of DNA extract per barcode were used as input for nanopore library preparation (as recommended by the manufacturer), depending on the input DNA concentration.

### Nanopore data basecalling and quality control

Basecalling, genome assembly, AMR gene detection and plasmid typing were conducted on a portable laptop with an 8 GB NVIDIA GeForce RTX 4070 GPU, 16 GB 5200 MHz RAM and an Intel i7-13800H CPU with 14 cores and 20 threads. The corresponding data analysis protocols are provided at https://github.com/ElaSrbrn/Carbapenem-resistant-Enterobacterales-Plasmid-clustering-pipeline. The raw nanopore data was basecalled using Dorado v5.0 and the SUP basecalling model (dna_r10.4.1_e8.2_400bps_sup@v5.0.0). We used Porechop v0.2.3 (https://github.com/rrwick/Porechop, accessed 28 June 2025) to trim the adapter sequences and filtered out low-quality reads (*Q*<9) and short sequences (<200 bases) using Nanofilt v2.8.0 (https://github.com/wdecoster/nanofilt, accessed 28 June 2025) [24]. Sequencing summaries were generated using Seqkit v2.10.0 (https://github.com/shenwei356/seqkit, accessed 28 June 2025) [25].

### Nanopore-based *de novo* assembly and genotyping

We generated *de novo* assemblies using Flye v2.9.5 [26] for high-quality long reads (nano-hq) followed by polishing with Medaka v2.0.1 (https://github.com/nanoporetech/medaka, accessed on 22 July 2025) in the bacterial mode (--bacteria). We assessed assembly coverage using SAMtools depth v1.18 [27], ensuring that all assemblies had a minimum median chromosomal coverage of 40× [28]. Contig circularity was visually assessed using Bandage v0.9.0 [29]. Bacterial genome quality metrics, including assembly completeness, contamination and GC content of the polished assemblies, were assessed using CheckM2 v1.1.0 using the uniref100.KO.1.dmnd reference database [30]. The chromosomes were reoriented to start at the *dna*A gene using dnaapler v1.2.0 [31].

We then analysed our polished *de novo* assemblies using the Pathogenwatch v2.3.1 platform (accessed 12 July 2025) for species identification, multi-locus sequence typing (MLST), and resistance gene detection [32]. Any discordance between Pathogenwatch and MALDI-TOF MS species calls was investigated with Kraken2 v2.1.4 [33] using default parameters and the National Center for Biotechnology Information (NCBI) nucleotide database (https://www.ncbi.nlm.nih.gov/nucleotide, accessed on 14 January 2025). Additionally, we confirmed the discordance in species identification with the *Citrobacter* spp. database hosted on PubMLST (accessed 12 July 2025) [34].

### Global phylogeny of publicly available *Citrobacter freundii* genomes

We next conducted phylogenetic analyses on publicly available *C. freundii* genomes to identify the species’ global diversity as a basis for our local clustering analyses. For this, complete *C. freundii* genomes were retrieved from the NCBI nucleotide database (https://www.ncbi.nlm.nih.gov/nucleotide, accessed 24 July 2025). Additionally, paired-end Illumina sequence data for *C. freundii* was obtained from the NCBI sequence read archive (SRA) (https://www.ncbi.nlm.nih.gov/sra, accessed 24 July 2025) for sequence data belonging to *C. freundii* using the ‘fasterq-dump’ tool within the SRA Toolkit v3.0.1-ubuntu64 (https://github.com/ncbi/sra-tools, accessed 28 July 2025), restricting searches to whole-genome sequencing, whole-genome amplification, whole-chromosome sequencing, clone-based, finishing or validation strategies. Raw Illumina sequence reads were *de novo* assembled using Shovill v1.1.0 (https://github.com/tseemann/shovill, accessed on 28 July 2025) with parameters set to estimate the genome size at 5 Mb, remove contiguous sequences (contigs) with a sequence coverage below 20-fold, and enable single-cell mode. Assembly metrics were assessed using QUAST v5.0.2 [35], with genomes removed if assemblies <4.3 or >6.2 Mb had N50 values <10,000 bp or sequencing depth <20×.

The resulting assemblies were input into a Genome Taxonomy Database Toolkit (GTDB-Tk) genome-based taxonomy (GTDB-Tk v2.1.1 with GTDB package R207_v2 [36]). Sequences within the genus *Citrobacter* and *Salmonella* (g_, genus) were extracted to construct a concatenated reference alignment of 120 bacterial marker genes. The taxonomic tree was inferred using maximum-likelihood approximation with FastTree v2.1.7 [37] under the WAG model [38] of protein evolution with gamma-distributed rate heterogeneity [39] (+ GAMMA). Genomes clustering outside of *C. freundii* species lineage were removed from downstream analyses. *In silico* MLST was done using MLST v2.23.0 (https://github.com/tseemann/mlst, accessed on 28 July 2025) with default settings to query the assemblies against the *Citrobacter* spp. typing database [40] hosted on PubMLST [34] (local database updated 19 March 2024). A total of 2,028 *C*. *freundii* genome assemblies [quality filtered (*n*=1,861); complete (*n*=167)] were aligned to create a core-genome alignment using Parsnp v1.7.4 [41], with the reference being the chromosome of CFTMDU (GenBank: CP151202), to identify SNPs. Resulting SNP alignments were used to reconstruct phylogenies. We used RaxML v8.2.12 [42] to build phylogenetic trees using the maximum-likelihood method with GTR-GAMMA correction (optimizing ten distinct, randomized maximum-parsimony trees). The phylogenetic trees were visualized using FigTree v1.4.4 (http://tree.bio.ed.ac.uk/software/figtree/, accessed 28 July 2025).

### Local genomic clustering of *C. freundii* sequence type (ST)91

We used the global *C. freundii* phylogeny to identify globally available C. *freundii* sequence type ST91 genomes, their closest phylogenetic outgroup, and investigate global delineation of the ST91 lineage. Genomic variants between all publicly available and our local ST91 genomes were identified using the SPANDx v4.0.4 pipeline [43], mapping reads to lineage-specific reference chromosomes. SNPs within regions of high-density clusters (≥3 SNPs in a 10 bps window), mobile genetic elements and predicted recombination sites (identified using Gubbins v3.3.5 [44]) were excluded. Sites were excluded if an SNP was called in regions with less than half or greater than threefold the average genome coverage on a genome-by-genome basis. This analysis defines a core genome as regions estimated to the nearest 100 bp with ≥95% coverage in all genomes, as calculated using the BEDTools v2.28.0 [45] coverageBed module within the SPANDx pipeline. The pairwise SNP distances were determined using snp-dist v0.6.3 (https://github.com/tseemann/snp-dists, accessed 22 July 2025). Maximum-parsimony trees were reconstructed from the orthologous biallelic core-genome SNP alignments using the heuristic search feature of PAUP v4.0a [46]. The pairwise SNP distance heatmap was visualized in R (v4.3.1) with the ggplot2, tidyr and dplyr packages.

### Carbapenemase gene detection and plasmid characterization

We identified contig-specific carbapenem resistance genes in our generated nanopore assemblies using AMRFinderPlus v4.0.3 (https://github.com/ncbi/amr, accessed 28 July 2025) with database version 2024-10-22.1 [47,48]. Carbapenemase-encoding contigs were functionally annotated with MOB-suite v3.1.8 [49]. The MOB-typer module [49] was applied to the contigs identified as plasmids to detect key mobility determinants and estimate the potential for horizontal gene transfer. We visualized the plasmid annotation using the mobileOG-db [50] integrated into ProkSee [51], with the carbapenemase positions confirmed using the CARD database [52].

### Plasmid similarity analysis and clustering

Plasmid similarity analysis and clustering were conducted for carbapenemase-encoding IncN plasmids that showed potential for interspecies transfer, as they were identified in different bacterial species within this study. To contextualize these plasmids within the publicly available global datasets, nucleotide sequence similarity was assessed using the Nucleotide blast search (blast+ v2.17 on https://blast.ncbi.nlm.nih.gov/Blast.cgi, accessed 10 November 2025) [53,55]. Searches were performed with megablast against the NCBI Core nucleotide blast (core_nt) database (updated on 5 October 2025) using default parameters, with the full-length IncN plasmid sequences as queries. blast alignments were ranked based on percentage identity and query coverage [56], with subject coverage estimates included to account for differences in query sequence length.

The subsequent plasmid clustering followed a stepwise approach beginning with pairwise MinHash (Mash)-based approximation of Jaccard distances for shared k-mers using MASH v1.4.5 [57]. We applied a Mash distance threshold of 0.001 to define highly similar plasmid sequences [5,58] and excluded those plasmids that did not fulfil this threshold criterion with any other plasmid from subsequent analyses. Structural relatedness of the remaining plasmids was then evaluated using the double-cut-and-join (DCJ)-indel model implemented in pling v1.0.1, applying a containment threshold of 0.3 (recommended for recent transmission events) and a default threshold of four DCJ and indel operations needed to transform one plasmid into another [58]. Additional exploratory linear comparative analyses of the assembled plasmids were performed using the ProkSee blastn module (v2.16.0) [Bibr R58][51].

## Results

### Species identification and antimicrobial resistance detection with established diagnostics

In total, 13 carbapenem-resistant *Citrobacter* spp. were isolated from patient-derived samples (*n*=10) and drain samples from either sinks or showers (*n*=3). Established laboratory diagnostics classified isolates as *Citrobacter farmerii* (*n*=2) and *C. freundii* complex (*n*=11) using MALDI-TOF MS and identified carbapenem resistance in all isolates using VITEK 2 (Methods; Table 1). Subsequent lateral flow assays identified six KPC-positive isolates, six isolates positive for both OXA-48-like and KPC carbapenemases and one isolate positive for OXA-48-like and VIM carbapenemases (Methods; Table 1). Notably, out of all rooms with confirmed patient occupancy by individuals carrying carbapenem-resistant *Citrobacter* spp. (samples 1–10), room D was the only room that yielded no CRE growth in drain samples. Follow-up drain sampling in March 2025 after identification of the CRE-positive samples 9 and 10 from room D again returned negative results for CRE growth in room D (Table 1).

**Table 1.** Bacterial species and antimicrobial resistance mechanism identifications across all isolates from patient [1,10] and drain (A–D) samples using established diagnostics and nanopore sequencing-based *de novo* assemblies For each sample, the sampling source, date and room are indicated. For nanopore sequencing, sequence types (ST) according to MLST and resistance gene location (chromosome- or plasmid-encoded with replicon types IncN or IncL/M) are additionally indicated in brackets. NA = not applicable.

Sample			Bacterial species identification		Antimicrobial resistance mechanism identification	
No.	Date	Room	Established diagnostic	Nanopore sequencing	Established diagnostic	Nanopore sequencing
1	02/2024	A	*C. freundii* complex	*Enterobacter mori*	KPC	KPC-2 (IncN)
2	03/2024	B and D	*C. freundii* complex	*C. freundii* (ST 91)	KPC OXA-48-like	KPC-2 (IncN), OXA-48 (IncL/M)
3	05/2024	C	*C. farmerii*	*C. farmerii* (ST 857)	KPC	KPC-2 (IncN)
4	06/2024	A and D	*C. freundii* complex	*C. freundii* (ST 91)	KPC OXA-48-like	KPC-2 (IncN), OXA-48 (IncL/M)
5	08/2024	B	*C. farmerii*	*C. farmerii* (ST 857)	KPC	KPC-2 (IncN)
6	09/2024	C	*C. freundii* complex	*Citrobacter portucalensis*	OXA-48-like VIM	OXA-48, VIM (chromosome)
7	09/2024	C	*C. freundii* complex	*C. freundii* (ST 91)	KPC OXA-48-like	KPC-2 (IncN), OXA-48 (IncL/M)
8	10/2024	A and B	*C. freundii* complex	*Citrobacter youngae*	KPC	KPC-2 (IncN)
9	03/2025	D	*C. freundii* complex	*C. freundii* (ST 91)	KPC OXA-48-like	KPC-2 (IncN), OXA-48 (IncL/M)
10	03/2025	D	*C. freundii* complex	*C. freundii* (ST 91)	KPC OXA-48-like	KPC-2 (IncN), Oxa-48 (IncL/M)
Drain A	10/2024	A	*C. freundii* complex	*Citrobacter portucalensis*	KPC	KPC-2 (IncN)
Drain B	10/2024	B	*C. freundii* complex	*C. freundii* (ST 18)	KPC	KPC-2 (IncN)
Drain C	10/2024	C	*C. freundii* complex	*C. freundii* (ST 91)	KPC OXA-48-like	KPC-2 (IncN), OXA-48 (IncL/M)
Drain D	10/2024	D	No growth	na	na	na

Global diversity and local clustering of *C. freundii* ST91.

### Nanopore sequencing and assembly metrics

Nanopore sequencing resulted in a mean N50 of >7 kb across all filtered sequencing reads (Methods), and an overall mean quality score ranging from 20.4 to 22.2 across all isolates (Table S2). All *de novo* assemblies of bacterial chromosomes and IncN plasmids exceeded the minimum median coverage of 40× (Table S3). Chromosomal assemblies ranged from 4.8 to 5.3 Mb in length. The GC content of the complete microbial genomes spanned from 0.51 to 0.55 (Table S4). All microbial genome assemblies were 100% complete and contamination ranged from 0.04 to 0.71%, indicating high-quality assemblies (Table S4). IncN plasmid assemblies ranged from 72 to 98 kb and GC content from 0.52 (sample 3 IncN) to 0.53 (the remaining IncNs). The IncL/M assemblies ranged from 72 kb (sample 4 IncL/M) to 63 kb (all remaining IncL/M plasmids) (Table S3).

### Bacterial species identification through genomic data analysis

Comparison of species identification between routine diagnostics and nanopore sequencing revealed important discrepancies and provided enhanced taxonomic resolution (Methods; Table 1). Nanopore sequencing confirmed the identification of two *C. farmerii* isolates initially classified by current routine diagnostics. However, the isolate from sample 1, initially identified as *C. freundii* complex, was reclassified as *Enterobacter mori* by all computational annotation tools, including Pathogenwatch, Kraken 2 and PubMLST (Methods; Table 1). For the remaining isolates identified within the *C. freundii* complex, long-read sequencing provided species-level resolution that routine diagnostics could not achieve: The isolates from sample 6 and drain sample A were identified as *Citrobacter portucalensis*, sample 9 was resolved to *Citrobacter youngae* and the remaining seven isolates to *C. freundii*, which are all part of the *C. freundii* complex (Methods; Table 1). MLST revealed that six of the seven *C. freundii* isolates belonged to the same high-risk *C. freundii* strain ST91 [59] (Methods; Table 1), indicating clonal relatedness.

To contextualize our ST91 isolates within the global diversity, we explored the relatedness of the six *C. freundii* ST91 isolates through global phylogenetic and subsequent local variant calling analyses. To correctly root the ST91 phylogeny, we first examined the overall population structure of *C. freundii* (Fig. S2) by including 2,028 publicly available *C. freundii* assemblies identified and filtered through our assembly-based taxonomic classification (Methods, Figs S2 and S3). Maximum-likelihood phylogenetic analysis identified 39 ST91 genomes from 13 countries spanning 2012 to 2024, comprising 33 human-derived isolates and four environmental samples from sources such as sewage, hospital drains, rivers, wastewater and from (for 2 genomes) unknown origin (Data S1). Clinical sample types showed diverse anatomical origins, with urine (*n* = 11) being the most common, followed by wound/abscess samples (*n* = 6). ST62 served as the closest outgroup to ST91, confirming that ST91 represents a well-defined lineage within the *C. freundii* species complex.

**Fig. 1.**
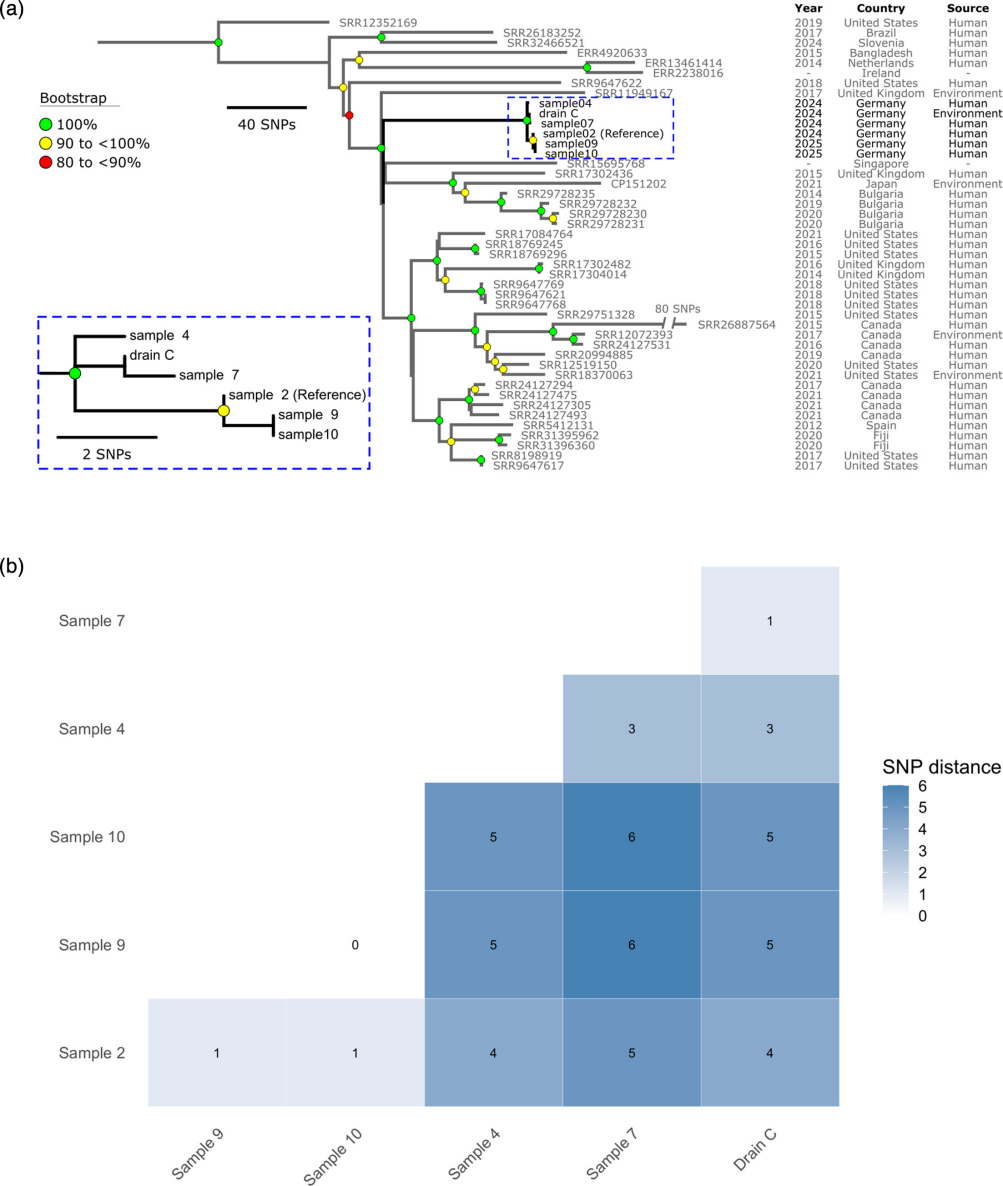
Comparison of *C. freundii* sequence type (ST)91 genomes based on SNPs. The 1,772 density-filtered non-recombinant orthologous biallelic SNPs were basecalled on a core-genome alignment of ~4,124,900 bp between the global *C. freundii* ST91 lineage (*n*=39) and the *C. freundii* ST91 genomes of this study (*n*=6), using sample 2 (GenBank accession number SAMN50449475) as a reference (Methods). (a) Maximum-parsimony phylogeny of all *C. freundii* ST91 genomes (consistency index of 1.0), using *C. freundii* ST62 as an outgroup (Methods). (b) Hierarchically clustered pairwise SNP-based distance matrix between the six *C. freundii* ST91 genomes of this study (Methods).

Integration of the six *C. freundii* ST91 bacterial chromosomes sequenced in this study to the 39 publicly available ST91 genomes revealed striking epidemiological patterns (Fig. 1a). The global ST91 phylogeny is genomically diverse, with pairwise SNP distances ranging from 0 to 396 SNPs [median = 153; interquartile range (IQR)= 116 to 189] (Data S1). In contrast, the six isolates from this study (2024 to 2025) form a highly clonal monophyletic cluster, with pairwise SNP distances ranging from 0 to 6 SNPs (median= 4; IQR=1.5 to 5 SNPs; Fig. 1b). Within this local cluster, pairwise SNP distances ranged from 0 to 6 SNPs (median=4; IQR=1.5 to 5 SNPs; Fig. 1b), indicating recent transmission or ongoing circulation within the hospital setting.

Within our cluster, isolates from samples 9 and 10 were genomically indistinguishable across the core genome (0 SNP), and the smallest pairwise SNP distance (1 SNP) was observed among the ST91 strains from samples 2, 9 and 10. Thus, the first ST91 index isolate of this local cluster (sample 2) formed a tight phylogenetic sub-branch with ST91 isolates from samples 9 and 10, reflecting their close relatedness (Fig. 1a). The ST91 from sample 7 also differed by a single SNP from drain C, further emphasizing the role of environmental reservoirs in resistance dynamics within the hospital setting. All observed SNP distances (Fig. 1b) fell well within established thresholds for defining epidemiologically linked clusters in *Enterobacterales* [60,61].

### Comprehensive antimicrobial resistance characterization through genomic data analysis

All carbapenemases detected by routine diagnostics were confirmed by genomic data analysis and the *de novo* assemblies additionally identified the subtype and genetic context of each carbapenemase (Methods; Table 1). All KPC-positive isolates across four different bacterial taxa carried the same KPC subtype KPC-2, which was located in discrete islands bordered by transposases (tnpA, tnpR) on a replicon type IncN plasmid of the AA552 MOB Cluster (Fig. S4A–C). The plasmids carried a complete conjugative transfer module characterized by a MOBF-type relaxase, an MBF_T-type mating pair formation system, and an identifiable origin of transfer (oriT), corresponding to the conserved tra/trfA region, confirming their classification as conjugative plasmids by MOB-suite [49].

OXA-48-like carbapenemases were confirmed as OXA-48 subtypes in all positive isolates. Notably, genetic context varied by bacterial species: OXA-48 was identified as chromosomally encoded in *C. portucalensis* (sample 6) in an IS-rich region flanked by a transposase gene tnpA and an integrase (int) (Fig. S5A), while all *C. freundii* ST91 isolates carried OXA-48 on conjugative IncL/M plasmids within a transposon-associated module next to the tnpA and integrase genes consistent with a TN1999-like configuration of OXA-48 (Table 1, Fig. S5B) [62,63].

Additional antibiotic resistance genes identified on the plasmids and chromosomes were consistent with the phenotypic resistance profiles observed for carbapenems and *β*-lactams (Data S2, interpreted according to the EUCAST guidelines [22]). Among these, only the respective carbapenemase genes provided sufficient explanation for the observed phenotypic carbapenem resistance (Data S2). While the IncN plasmid carried multiple antibiotic resistance determinants, including KPC-2, the IncL/M plasmid harboured only OXA-48 (Data S2 and Fig. S5B). Additional conjugative plasmids were identified in isolates from samples 2, 6, 7, 8, 9 and 10 and drain sample C (Data S2), albeit without any encoded antibiotic resistance genes.

### Plasmid clustering reveals complex transmission networks

Given the multi-species distribution of KPC-2-carrying IncN plasmids across different environmental and clinical sources, we focused plasmid clustering analyses on the IncN plasmids to better understand their dynamics (Methods, Table 1). The top ten blastn alignments of the IncN plasmids against the NCBI core_nt database revealed high sequence identity and coverage with previously reported KPC-2–carrying IncN plasmids from diverse hosts, including *Enterobacter* spp., *Klebsiella* spp., *Citrobacter* spp. and *Escherichia coli* (Data S3) [7,64, 65].

Mash distance analysis revealed distinct clustering patterns (Methods), with plasmids from sample 3 (*C. farmerii*) and drain sample B (*C. freundii* ST 18) showing a pairwise distance of >0.001 with all other IncN plasmids and were therefore excluded from further plasmid clustering analyses (Table S5). The IncN plasmid from drain sample B was at least 10 kb longer than the other IncN plasmids (Table S3), which is partially explained by an additional region enriched in mobility- and stability-associated genes, including multiple transposases (tnpA) and integrases (intM), consistent with the insertion of a composite mobile element forming a mosaic island, when compared to ST91 IncN plasmids (Fig. S6A). The IncN plasmid from sample 3 (length of 72 kb) was shorter than the ST91 IncN plasmids and lacked the aac(3)-IId aminoglycoside resistance gene (Fig. S6B).

**Fig. 2.**
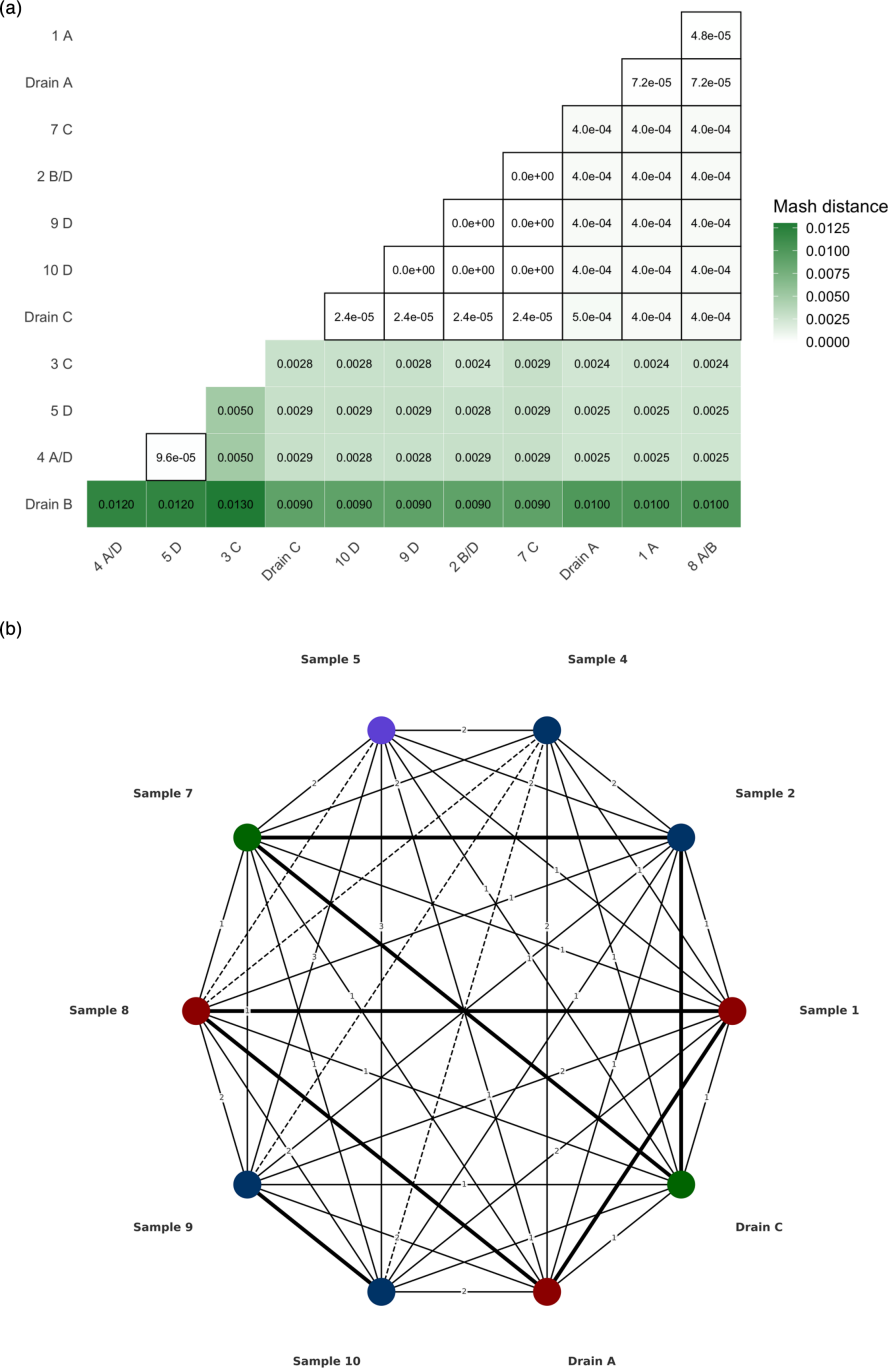
Stepwise clustering analyses of IncN plasmids based on sequence (**a**) and structural (**b**) similarity. (a) Heatmap of pairwise Mash distances between all IncN plasmids of this study (see Table 1). Mash distances below the threshold of 0.001, which indicate plasmid pairs with high sequence similarity, are highlighted by cell borders (Methods). (b) DCJ-indel-based clustering network of the IncN plasmids with at least one high pairwise sequence similarity match (sample 3 and drain B are excluded). Nodes represent individual samples and are coloured according to their room location (red, room A; violet, room B; green, room C; blue, room D; see Table S6). Edges represent pairwise DCJ-indel distances: edges with distances of 1–3 are labelled with their DCJ-indel value, edges with a distance of zero are shown as bold solid lines and edges with distances ≥4 are shown as dashed lines.

All remaining plasmids that fulfilled the Mash distance requirement (<0.001) also satisfied DCJ-indel clustering criteria (DCJ-indel distance ≤4; Methods; Fig. 2b, Table S6). Within this IncN plasmid cluster, the closest subcluster based on the Mash distance was observed between sample 1 (*E. mori*), sample 8 (*C. youngae*) and drain sample A (*C. portucalensis*); as all these samples were associated with room A, this might suggest room-specific plasmid circulation (Tables 1 and S5, Figs 2a and S4a). The IncN plasmids of five of the six *C. freundii* ST91 strains (found in samples 2, 7, 9 and 10 and drain sample C) further formed a subcluster with very low Mash distances (Figs 2a and S4b). On the other hand, the IncN plasmid of the *C. freundii* ST91 strain from sample 4 was 10 kb longer than the other ST91 IncN plasmids (Table S3) and of similar length to the IncN plasmid of *C. farmerii* from sample 5, with an additional Tn3 transposon insertion partially accounting for this length difference (Fig. S4c). This length concordance between the IncN plasmids from samples 4 and 5 was associated with a low pairwise Mash distance between the two plasmids (Figs 2a and S4c, Table S5), whereas the DCJ-indel distance suggests some degree of structural divergence (Table S6, Fig. S4c), highlighting a discordance between Mash and DCJ-indel distance measurements.

## Discussion

We highlight the relevance of long-read whole-genome sequencing for uncovering bacterial strain- and plasmid-mediated carbapenem-resistance dynamics between patient and environmental reservoirs in the hospital, which would otherwise be missed by established diagnostics. In our example, routine culture and susceptibility testing flagged a rise in carbapenem-resistant *Citrobacter* spp. in an internal medicine ward over a 13-month period. However, the information provided by these routine diagnostic tools could not discriminate whether this trend reflected (i) a clonal spread of a single *Citrobacter* strain, (ii) a horizontal transfer of distinct plasmids, (iii) a gain or loss of OXA-48 within a shared KPC-encoding plasmid or (iv) an independent accumulation of unrelated carbapenem-resistant *Citrobacter* spp. In contrast to routine diagnostic procedures, whole-genome sequencing by long-read nanopore technology provided higher-resolution taxonomic profiles, allowing us to exclude unrelated subspecies or strains from further downstream clonal analyses and to accurately group those with greater chromosomal similarity [66]. By retrospectively generating *de novo* assemblies from these bacterial isolates, we were able to resolve resistance dynamics at both the chromosomal and plasmid levels, and we showed that the accumulation of CREs was a mixture of a circulating *C. freundii* ST91 and plasmid-mediated resistance spread across bacterial species and the hospital environment.

More specifically, we identified a *C. freundii* ST91 cluster comprising six isolates from patient screening samples and one from a drain sample. Notably, this cluster was genetically distinct from other global *C. freundii* ST91. Within our hospital cluster itself, we found two identical isolates collected within the same month (samples 9 and 10), differing by only one SNP from the index ST91 isolate (sample 2), which had been sampled 12 months earlier in the same room. As this SNP-based distance is below the expected core-genome point-mutation rate in actively replicating *Enterobacterales* [61,67], such genomic stasis could be indicative of a low-replication reservoir rather than continuous spread over more than 12 months. One plausible source for this would be environmental biofilms in the hospital setting as found in drains, where CREs embedded in biofilms replicate slowly, accrue few mutations, and can intermittently slough off biofilm fragments that then reseed with nearly identical clones [11,12, 68]. However, while the other rooms implicated in this study were CRE-positive, even repeated drain cultures from this index room remained negative. This finding might highlight limitations of culture-based surveillance for dormant biofilms, where an extension of the liquid culture enrichment beyond 48 h or direct metagenomic sequencing of environmental samples could potentially increase sensitivity. Alternatively, the environmental reservoir might have been located outside of the drain system, which emphasizes the need for more extensive environmental surveillance in the clinical setting. Independent of the scenario, the fact that the index ST91 isolate formed a subcluster with isolates from samples collected 12 months later and is more distantly related to the intermediate ST91 isolates suggests that the true root of the bacterial transmission chain in the internal medicine ward might have been missed. This highlights the common challenges of retrospectively inferring transmission events based on a limited sample size sampled across long time intervals [69].

Beyond chromosomal insights, long-read nanopore sequencing data generated near-complete *de novo* assemblies of plasmids, clarifying whether carbapenem resistance genes were encoded on a plasmid or chromosome, and uncovering the relatedness of plasmids between bacterial species and sampling sites. These *de novo* assemblies identified the role of a KPC-2–carrying IncN plasmid in the described rise of carbapenem-resistant isolates. The sequence-level and structural comparisons of the IncN assemblies revealed evidence of interspecies plasmid transfer across four bacterial taxa and of dissemination between patient and environmental reservoirs over 8 months, thereby allowing us to confirm (or rule out) potential epidemiological links. The interpretation of plasmid dynamics, however, requires caution since plasmids show high genomic plasticity and lack a defined molecular clock (such as SNP-based distance for bacterial chromosomes) to infer plasmid relatedness. Thus, any threshold-based inference must still be carefully cross-checked against the epidemiological context before conclusions are drawn. To mitigate this uncertainty, we assessed plasmid relatedness with two complementary metrics: sequence similarity (Mash distance) and structural similarity (DCJ-indel distance). This approach captures the high plasticity of plasmids that may undergo substantial structural changes with minimal changes in sequence content and vice versa [58]. Furthermore, we applied stringent cut-offs as recommended in previous studies [5,58] to minimize the risk of including false positive links between plasmids. However, care must be taken when comparing plasmids of different lengths, since this can impact the sequence-based Mash distance, which was used as a first metric for the stepwise plasmid clustering approach [5]. Such length differences between plasmids can impact sequence-based Mash and structure-based DCJ-indel distances differently, highlighting the complexity of plasmid clustering analyses.

Our results highlight that plasmids can readily cross bacterial species and reservoir boundaries over extended periods within the hospital setting, with direct implications for clinical surveillance and infection control strategies. We suggest that clinical surveillance efforts should move beyond single-species analyses and explicitly account for the fluid and modular evolution of plasmids. The likely exchange of IncN plasmids between environmental and patient-derived isolates might suggest that biofilms act as persistent reservoirs of carbapenemase-encoding plasmids in clinical settings. Integrating plasmid genomics into routine surveillance could therefore provide actionable insights into local carbapenem resistance dynamics – for example, by identifying high-risk plasmid lineages and their associated resistance elements to guide targeted containment measures such as intensified disinfection of reservoirs with confirmed plasmid persistence.

However, a few additional limitations should be noted. The analysis is based on a small number of isolates (*n*=13), and the dataset was assembled retrospectively, which may limit generalizability. Larger prospective studies – ideally including sampling across multiple sites and time periods – would help validate the observed patterns and clarify their clinical relevance across bacterial populations and circulating lineages in the hospital environment.

Overall, we provide laboratory and computational guidelines for bacterial whole-genome clustering and plasmid-mediated resistance dynamic detection through nanopore whole-genome sequencing in clinical settings. Our findings reveal the limitations of relying solely on routine culture-based diagnostics to capture the complexity of carbapenem resistance dynamics and underscore the value of nanopore sequencing in resolving both chromosomal and plasmid-level relationships between patient and environmental reservoirs in the clinical setting.

## Supplementary material

10.1099/mgen.0.001644 Uncited Supplementary Material 1.

10.1099/mgen.0.001644 Uncited Supplementary Material 2.

10.1099/mgen.0.001644 Uncited Supplementary Material 3.

10.1099/mgen.0.001644 Uncited Supplementary Material 4.

## Abbreviations

Contig: contiguous sequence
CRE: carbapenem-resistant *Enterobacterales*
DCJ: double-cut-and-join
ESBL: extended-spectrum beta-lactamase
EUCAST: European Committee on Antimicrobial Susceptibility Testing
GTDB-Tk: Genome Taxonomy Database Toolkit
IPC: infection prevention and control
IQR: interquartile range
KPC: *Klebsiella pneumoniae* carbapenemase
MLST: multi-locus sequence typing
NCBI: National Center for Biotechnology Information
SRA: Sequence Read Archive
ST: sequence type
