# Supplementary material for: Resolving plasmid-encoded carbapenem resistance dynamics and reservoirs in a hospital setting through nanopore sequencing
Source: Microb Genom. 2026 Feb 12;12(2):001644. doi: 10.1099/mgen.0.001644 (PMC12900256; doi:10.1099/mgen.0.001644)
Supplement: Uncited Supplementary Material 1. [file mgen-12-01644-s001.pdf]

Resolving plasmid-encoded carbapenem resistance dynamics and reservoirs in a hospital setting through nanopore sequencing

Supplementary information

Ela Sauerborn[1,2,3*], Rhys T. White[4], Anna-Lena Kalteis[3,5], Daniel Gygax[1,2,6], Ebenezer Foster-Nyarko[7], Nina Wantia[3], Friedemann Gebhardt[3,5], Lara Urban[1,2,8*]

[1]Helmholtz AI-Institute, Helmholtz Center Munich, Germany,

[2]Helmholtz Pioneer Campus, Helmholtz Center Munich, Germany

[3]Institute of Medical Microbiology, Immunology and Hygiene, Department of Preclinical Medicine, Technical University Munich, Germany

[4] New Zealand Institute for Public Health and Forensic Science, Health Security, Porirua, New Zealand

[5]Technical University of Munich, TUM School of Medicine and Health, TUM University Hospital, Munich, Germany

[6]RIFCON GmbH, Hirschberg an der Bergstraße, Germany

[7]Department of Infection Biology, London School of Hygiene & Tropical Medicine, London, UK

[8]Institute of Food Safety and Hygiene, University of Zurich, Switzerland

*corresponding authors: elasauerborn@gmail.com.de; lara.h.urban@gmail.com

This file includes

- Supplementary Figures 1-6
- Supplementary Tables 1-6

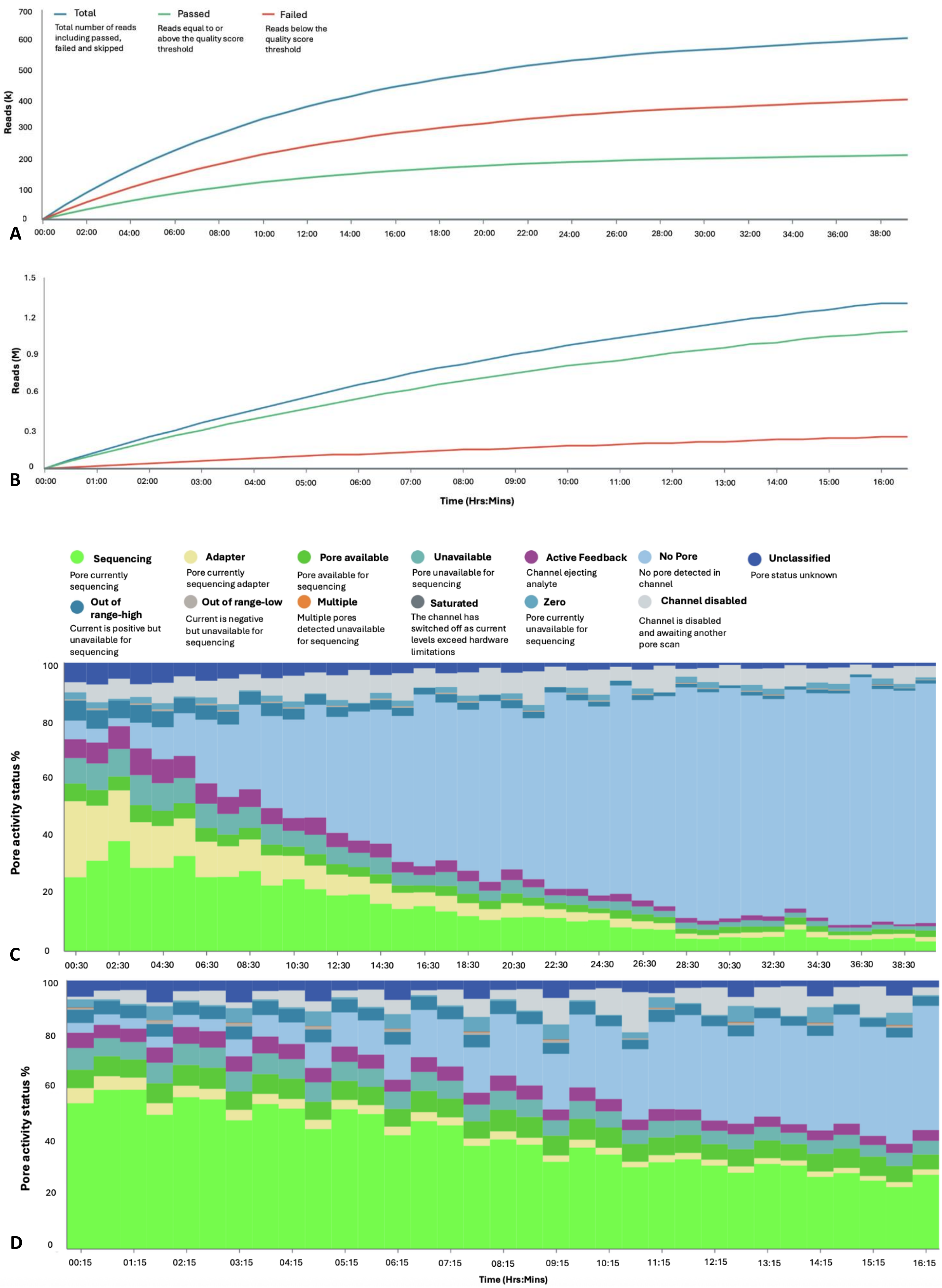

Supplementary Fig. 1. Nanopore sequencing performance comparison without (A, C) and with (B, D) RNase treatment of the same DNA extracts (Table S1; Methods). (A, B) Sequencing read throughput over time, measured by the number of total, passed, and failed reads over sequencing time. Reads are classified as failed if they have a quality score below 8, based on FAST basecalling. Counts area

shown in thousands (k) and millions (M). (C, D) Nanopore occupancy over sequencing time; actively sequencing pores are colored in bright green, unavailable pores are colored in bright blue and pores sequencing adapters are highlighted in yellow (adapted from the sequencing reports generated by the Oxford Nanopore Technologies' MinKNOW software v24.06.16).

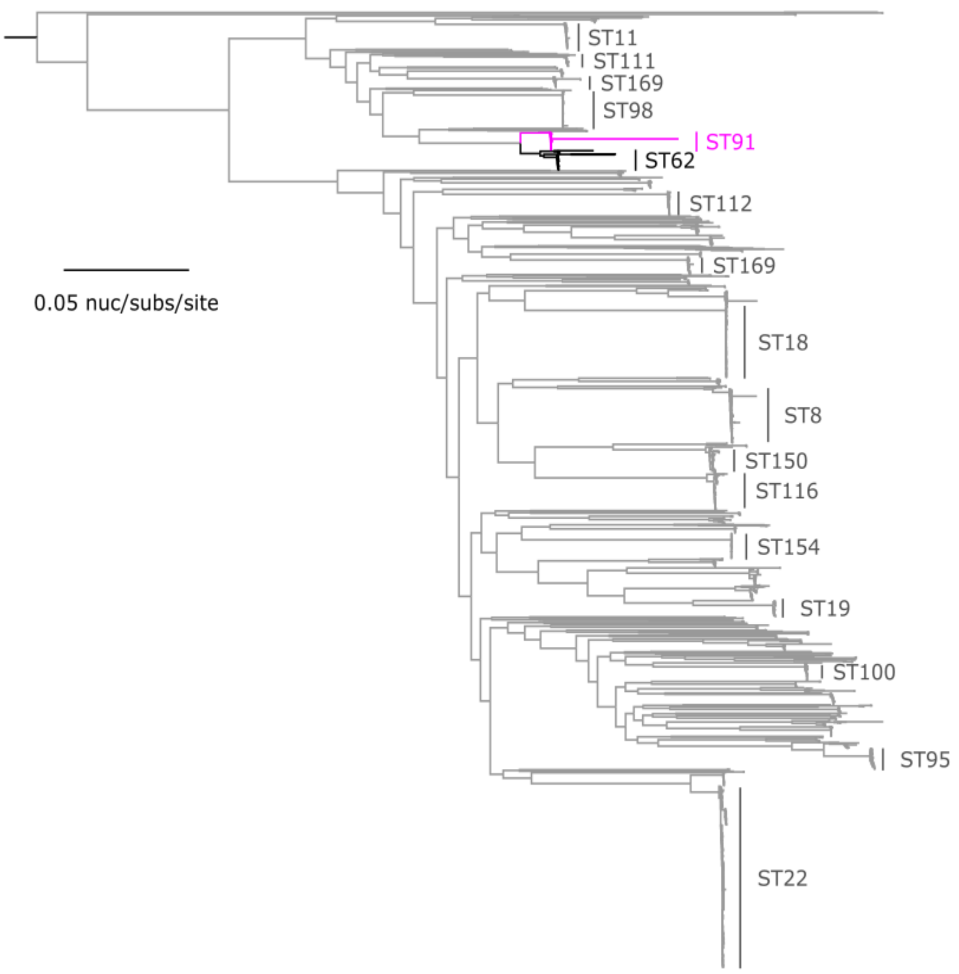

Supplementary Fig. 2. **Maximum-likelihood phylogeny of *Citrobacter freundii*.** The phylogeny was inferred from 121,511 core-genome single-nucleotide polymorphisms (SNPs) from 2,028 assembled (publicly available) genomes. SNPs were derived from a core-genome alignment of 1,431,649 bp and are called against the 5,093,232 bp complete chromosome of CFTMDU (GenBank: CP151202). A sequence type (ST) comprised of >10 genomes is labelled. The phylogeny was rooted at the midpoint.

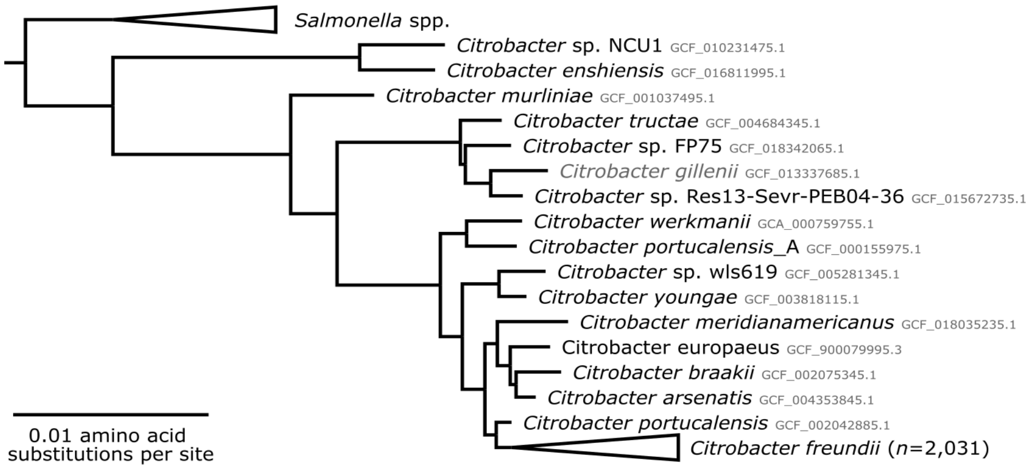

Supplementary Fig. 3 Taxonomic identification of publicly available *C. freundii* complex genomes. Retrieved from the NCBI nucleotide database and sequence read archive using the GTDB-Tk taxonomic identification tool. The taxonomic tree is based on a maximum-likelihood approximation of protein evolution assuming a gamma-distributed rate heterogeneity. Only genomes that clustered within the *C. freundii* species lineage were included in downstream analyses.

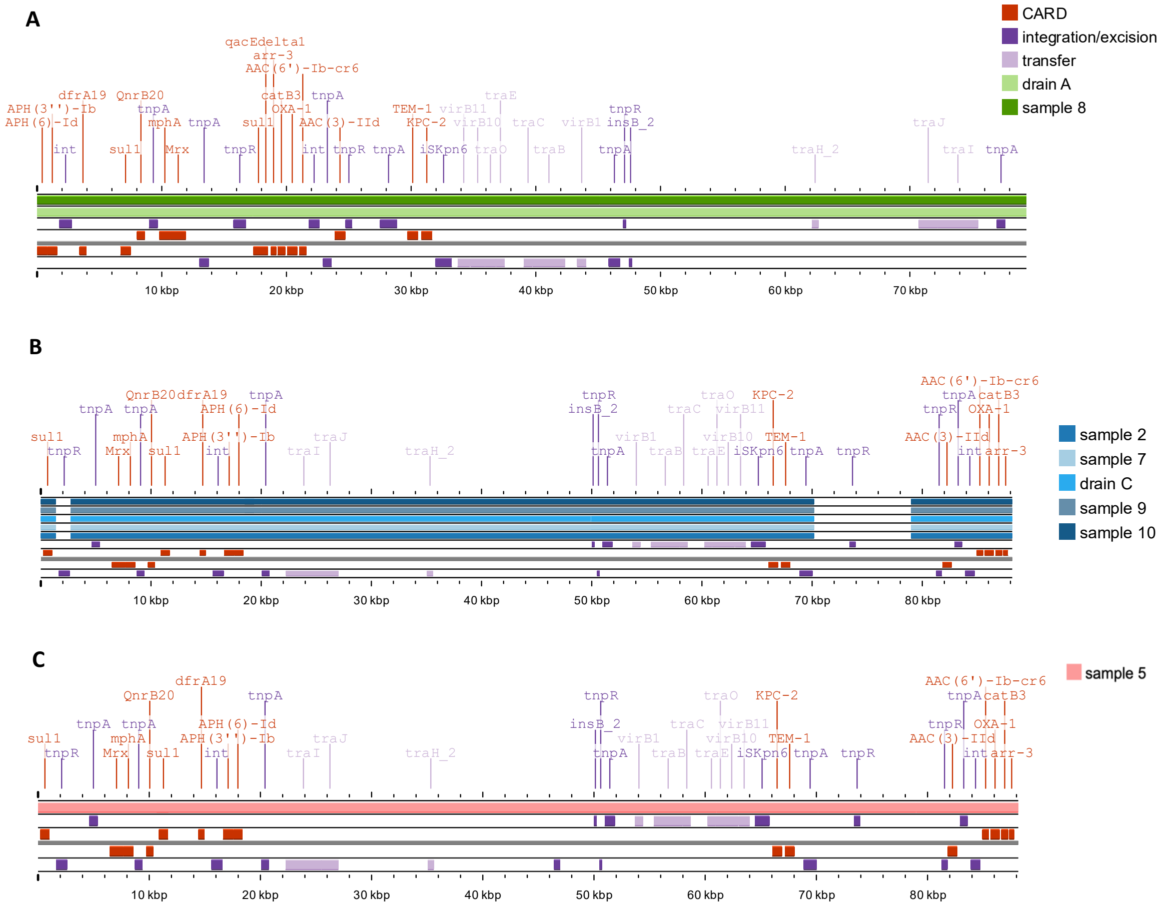

Supplementary Fig. 4. Blastn alignments (v2.16.0) of IncN plasmids in grey (A: sample 1, B and C: sample 4) meeting the Mash distance threshold of <0.001 with at least one other IncN plasmid. In each panel, the respective chronologically first sample (sample 1 in A, sample 4 in C) or longest sequence (sample 4 in B) are used as reference and shown in grey. Integration and excision annotation based on the mobileOG-db v1.1.3 is highlighted in dark purple, and transfer-associated genes are shown in light purple and antibiotic resistance genes annotated by CARD RGI v6.0.3 are shown in red. Created with ProkSee.com; NA annotations were removed manually to improve visual clarity.

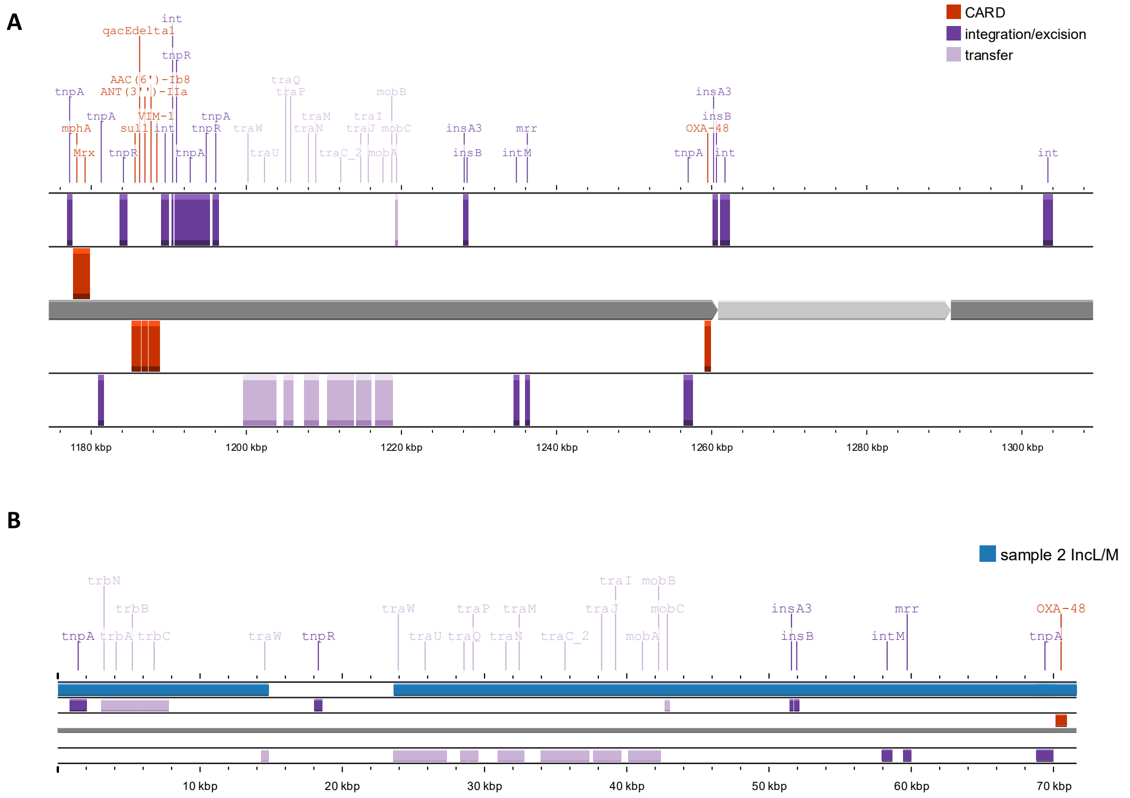

Supplementary Fig. 5. Genetic context of OXA-48 in plasmid- and chromosome-encoded configurations. **A.** Chromosomal excerpt from sample 6 in grey showing the OXA-48-encoding region adjacent to tnpA transposase. Additional mobile-element markers (insA3, insB, int) are located on a neighbouring chromosomal contig. **B.** Comparative visualization of IncL/M plasmids from sample 4 (grey) and sample 2 (blue). Both plasmids carry the blaOXA-48- gene flanked by a tnpA transposase. The longer plasmid from sample 4 (71,622 bp) includes an additional tnpR insertion, accounting for part of its increased length compared to the 62,813 bp IncL/M backbone from sample 2.Integration and excision annotation based on the mobileOG-db v1.1.3 is highlighted in dark purple, and transfer-associated genes are shown in light purple and antibiotic resistance genes annotated by CARD RGI v6.0.3 are shown in red. Created with ProkSee.com; NA annotations were removed manually to improve visual clarity.

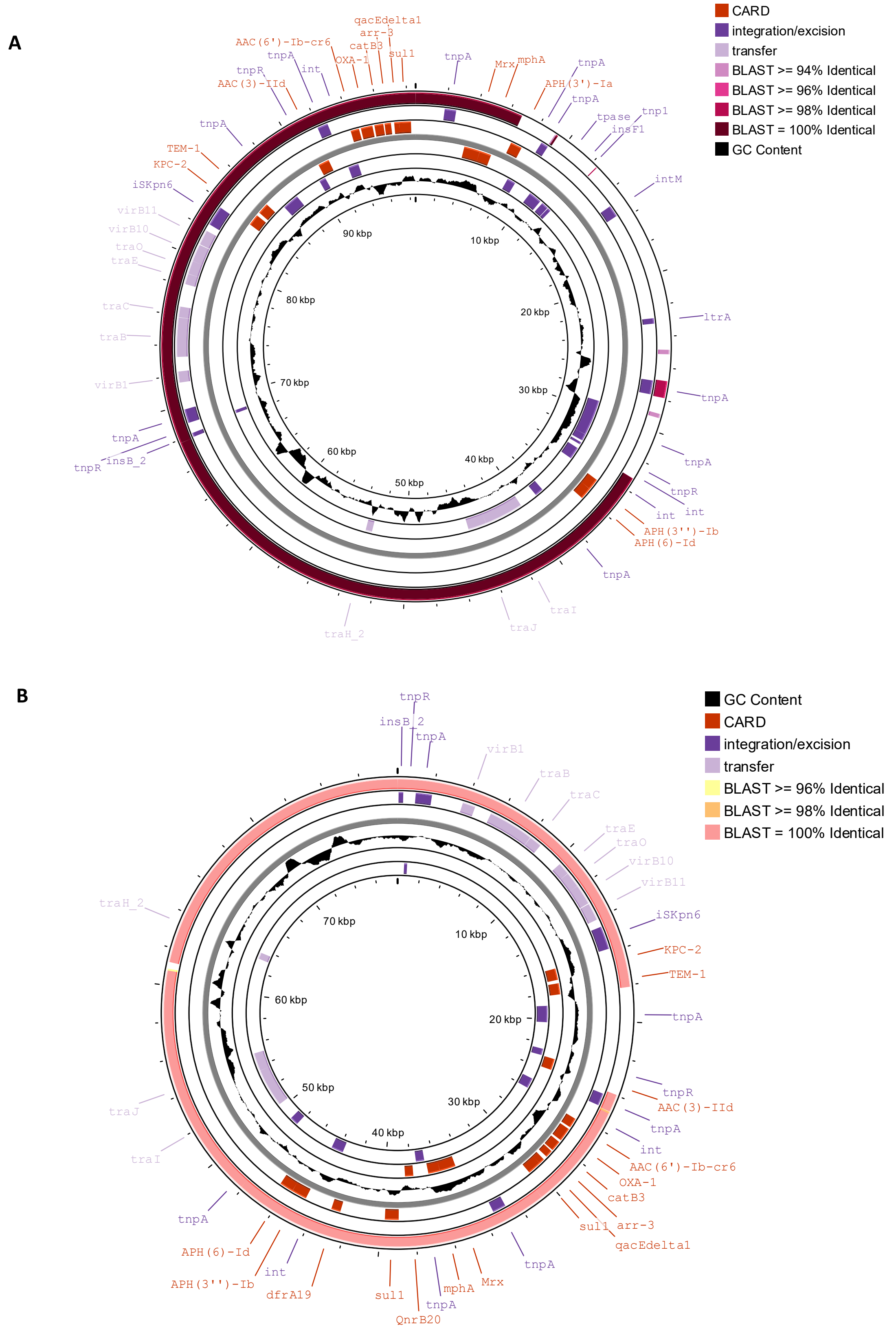

Supplementary Fig. 6 Comparative visualization of the two IncN plasmids excluded from plasmid clustering based on their Mash distance (>0.001). GC content skews in the respective reference sequence are shown as deviations from 50% in black. Integration and excision annotation based on the mobileOG-db v1.1.3 is highlighted in dark purple, and transfer-associated genes are shown in light

purple and antibiotic resistance genes annotated by CARD RGI v6.0.3 are shown in red. The figure was generated with ProkSee.com (GC content tool v1.0.3; BLASTN v2.16.0). NA annotations were removed manually to improve visual clarity. **A.** IncN plasmid from drain B (grey, reference) against the drain C IncN plasmid as ST91 IncN plasmid representative (colored according to BLASTN score, pink to purple gradient). **B.** IncN plasmid from drain C as ST91 IncN plasmid representative (grey, reference) against the IncN plasmid from sample 3 (colored according to BLASTN score, pink to orange gradient).

Supplementary Table 1. DNA extraction and nanopore sequencing metrics of nine DNA extracts (two barcodes per extracts) that were sequenced without or with post hoc RNase treatment (Methods). Comparison of Nanodrop nucleotide and Qubit DNA concentrations, and their relative difference (measured as [Nanodrop (ng/µl)-Qubit (ng/µl)]/Nanodrop (ng/µl)) prior to upconcentration. Up to 200ng of DNA per barcode were used as input for nanopore library preparation (as recommended by the manufacturer), or as much DNA as available in 10µl of DNA extract after purification and upconcentration. Nanopore sequencing results are presented in total yield (number of Mbases) and percentage of passed bases (after application of a FAST-basecalling quality score filter of 8).

Barcodes	RNase treatment	Nanodrop (ng/µl)	Qubit (ng/µl)	[Nanodrop-Qubit]/Nanodrop	Input DNA (ng)	Yield (Mb)	Passed bases (%)
**1-2**	No	598.8	17	0.97	400	11.97	13.95
	Yes	1.9	1.7	0.11	240	414.2	96.10
**3-4**	No	674.2	62	0.91	400	82.23	77.56
	Yes	3.6	1.8	0.5	106	498.1	95.65
**5-6**	No	1484	35	0.98	400	145.12	81.35
	Yes	5.0	1,6	0.68	164	173.62	91.69
**7-8**	No	532.8	20	0.96	400	37.27	14.30
	Yes	1.5	1,3	0.13	148	70.35	87.05
**9-10**	No	655.0	36	0.95	400	44.65	47.55
	Yes	1.8	1,1	0.39	180	293.75	95.75
**11-12**	No	520.3	31	0.94	400	24.11	11.75
	Yes	2.5	1,8	0.28	194	333.36	87.85
**13-14**	No	228.2	35	0.85	400	17.95	26.65
	Yes	2.2	1,4	0.36	188	204.10	89.55
**15-16**	No	140.4	26	0.81	400	25.80	63.0
	Yes	5.1	4,3	0.16	400	379.89	93.75
**17-18**	No	374.1	32	0.91	400	19.98	13.3
	Yes	1.5	1.4	0.07	188	18.04	94.0

Supplementary Table 2. Sequencing summary of the filtered and basecalled nanopore sequencing data across all isolates, including total number of reads, read-level N50, total number of bases, as well as mean base-level quality score, and percentage of bases with a quality score above 20.

Sample	# reads	read N50	# bases	mean Q-score	Q20 (%)
**1**	130,785	5,801	456,322,626	22.16	94.85
**2**	166,338	5,228	496,481,848	21.43	93.91
**3**	99,683	6,402	358,492,576	20.64	92.26
**4**	189,570	6,897	690,118,598	20.98	93.03
**5**	500,513	6,328	1,769,558,787	21.4	93.74
**6**	128,310	9,480	593,636,606	21.0	93.17
**7**	130,553	4,272	321,674,712	20.42	92.92
**8**	265,115	10,340	1,406,718,773	21.84	94.36
**9**	269,725	6,665	944,512,723	21.21	93.72
**10**	180,221	8,133	713,718,257	21.58	93.98
**Drain A**	322,607	8,139	1,353,038,408	21.83	94.26
**Drain B**	113,529	5,573	382,614,256	21.40	94.00
**Drain C**	182,033	8,897	827,156,867	21.08	93.22

Supplementary Table 3. Assembly metrics overview of each isolates' bacterial chromosome and (if present) IncN and IncL/M plasmid, including contig length, median coverage, and contig circularity. All plasmids were confirmed to be circular.

Sample	Chromosome			IncN plasmid		IncL/M plasmid	
	Contig length (b)	Median coverage	Circular	Contig length (b)	Median coverage	Contig length (b)	Median coverage
**1**	4,904,886	85	✓	79,348	162	NA	NA
**2**	5,248,615	90	✓	78,021	156	62,813	121
**3**	5,134,109	59	✓	72,036	132	Na	NA
**4**	5,247,584	58	✓	88,158	41	71,622	87
**5**	5,131,833	314	✓	88,157	273	NA	NA
**6**	5,303,591	46	X	NA	NA	NA	NA
**7**	5,248,812	109	✓	78,051	179	62,812	81
**8**	4,860,193	282	✓	79,348	386	NA	NA
**9**	5,248,611	107	✓	77,380	214	62,812	143
**10**	5,348,650	70	✓	77,391	85	62,812	108
**Drain A**	4,794,503	275	✓	79,349	263	NA	NA
**Drain B**	5,020,617	57	✓	98,563	91	NA	NA
**Drain C**	5,249,966	152	✓	78,032	253	62,812	132

Supplementary Table 4. Summary of quality control metrics for polished genome assemblies of all isolates showing estimated completeness, contamination, and GC content as determined by CheckM2.

Sample	Genome assembly quality metric		
	Completeness (%)	Contamination (%)	GC-Content
**1**	100	0.04	0.55
**2**	100	0.06	0.52
**3**	100	0.17	0.53
**4**	100	0.16	0.52
**5**	100	0.11	0.53
**6**	100	0.5	0.51
**7**	100	0.06	0.52
**8**	100	0.07	0.52
**9**	100	0.06	0.52
**10**	100	0.07	0.52
**Drain A**	100	0.05	0.52
**Drain B**	100	0.71	0.52
**Drain C**	100	0.06	0.52

Supplementary Table 5. Pairwise Mash distances of all IncN plasmids detected in bacterial isolates across samples. Pairwise distances at a threshold of >0.001 are marked in grey (Methods).

**Sample & Room**	**2 B/D**	**3 C**	**4 A/D**	**5 D**	**7 C**	**8 A/B**	**9 D**	**10 D**	**Drain A**	**Drain B**	**Drain C**
**1 A**	0.0004	0.0024	0.0025	0.0025	0.0004	**4.77e-05**	0.0004	0.0004	**7.16e-05**	0.010	0.0004
**2 B/D**	x	0.0029	.0029	0.0028	**0**	0.0004	**0**	**0**	0.0004	0.009	**2.39e-05**
**3 C**	0.0024	x	0.005	0.005	0.0029	0.0024	0.0028	0.0028	0.0024	0.013	0.0028
**4 A/D**	.0029	0.005	x	**9.55e-05**	0.0029	0.0025	0.0028	0.0028	0.0025	0.012	0.0029
**5 D**	0.0028	0.005	9.55e-05	x	0,0029	0.0025	0.0029	0.0029	0,0025	0.012	0,0029
**7 C**	0	0.0029	0.0029	0,0029	x	0.0004	0	0	0.0004	0.009	**2.38 e-05**
**8 A/B**	0.0004	0.0024	0.0025	0.0025	0.0004	x	0.0004	0.0004	**7.16e-05**	0.010	0.0004
**9 D**	0	0.0028	0.0028	0.0029	0	0.0004	x	**0**	0.0004	0.009	**2.38e-05**
**10 D**	0	0.0028	0.0028	0.0029	0	0.0004	**0**	x	0.0004	0.009	**2.38e-05**
**Drain A**	0.0004	0.0024	0,0025	0,0025	0.0004	**7.16e-05**	0.0004	0.0004	x	0.010	0.0005
**Drain B**	0.009	0.013	0.012	0.012	0.009	0.010	0.009	0.009	0.010	x	0.009
**Drain C**	**2.39e-05**	0.0028	0,0029	0,0029	**2.38e-05**	0.0004	**2.38e-05**	**2.38e-05**	0.0005	0.009	x

Supplementary Table 6. Contingency table of DCJ-indel distances between all IncN plasmids including sample 3 and drain B that were excluded from downstream clustering analyses. Isolates are described by their sample number and associated hospital rooms. The plasmid pairs that did not meet the Mash threshold of <0.001 are marked in grey (Methods).

Sample & Room	2 B,D	3 C	4 A, D	5 D	7 C	8 B	9 D	10 D	Drain A	Drain B	Drain C
**1 A**	1	3	1	1	1	0	2	2	0	1	1
**2 B,D**	x	4	2	2	0	1	1	1	1	2	0
**3 C**	4	x	3	4	4	7	5	5	3	4	3
**4 A,D**	2	3	x	2	2	4	5	5	2	2	1
**5 D**	2	4	2	x	2	4	3	3	1	2	1
**7 C**	0	4	2	2	x	1	1	1	1	2	0
**8 B**	1	7	4	4	1	x	2	2	0	3	1
**9 D**	1	5	5	3	1	2	x	0	2	3	1
**10 D**	1	5	5	3	1	2	0	x	2	3	1
**Drain A**	1	3	2	1	1	0	2	2	x	3	1
**Drain B**	2	4	2	2	2	3	3	3	3	x	2
**Drain C**	0	4	2	2	0	1	1	1	1	3	x
